# Is There a Need to Modify Existing Coping Scales to Include Using Electronic Media for Coping in Young People?

**DOI:** 10.3389/fped.2014.00127

**Published:** 2014-11-17

**Authors:** Marie Leiner, Beverley Argus-Calvo, Jesus Peinado, Liz Keller, Dan I. Blunk

**Affiliations:** ^1^Department of Pediatrics, Texas Tech University Health Sciences Center, El Paso, TX, USA; ^2^Department of Educational Psychology and Special Services, The University of Texas at El Paso, El Paso, TX, USA; ^3^Department of Medical Education, Texas Tech University Health Sciences Center, El Paso, TX, USA

**Keywords:** youth coping mechanisms, Stress, Psychological, assessment, media devices, technology

Children and adolescents spend an average of 7 h a day using electronic media which include television, radio, cellphones, computers, and handheld devices (1); it would be naïve to think that time spent on these devices has no effects (2). Indeed, it is clear that media has an effect on all of us, especially, youth who have grown up in a world surrounded by electronic devices (3–5). Decades of study on media use indicate a profound effect on the lives, health, and well being of youth (1). In particular, findings from a large number of studies, though often subject to controversy, link media use to psychosocial (6, 7), behavioral, and health problems (8–10) in children and youth. Potential positive effects of media use have been less studied. Media entertains, teaches, is a tool for communication, and may also be used as a means to cope with stress. We propose a need for the development of a novel assessment scale that will facilitate studies of the role of electronic media as a youth coping strategy.

Few studies have addressed the use of electronic media as a stress-coping mechanism. The findings that exist are mainly subjective, focus on its effects on socialization, or consider it a contributor to stress (11, 12). However, it has been reported that electronic media consumption among youth represents a form of mood management or social compensation (13). Online communication has been shown to benefit self-esteem and increase perceived social support (14). By allowing youth a way to communicate with friends and to present themselves to others, electronic media provides an opportunity for self-disclosure and identity experimentation (14, 15). Thus, moderate use has been shown to have a positive effect on social skills, interpersonal communication, and activities (16, 17). Some studies, for example, have found that instant messaging can provide emotional relief, allowing users to vent negative emotions while providing the possibility to receive social support and advice (18). Similarly, texting allows users to take their mind off of the present (19) and is often used by youth as a way of coping with stressful situations. Finally, studies of online support groups have found that they give youth an opportunity to show empathy and emotional relief (20, 21). By contrast, watching television (22), though one of the most common choices for coping with psychological stress, appears to be one of the least successful (23).

When evaluating findings from previous studies, it is important to keep in mind that electronic media was not as relevant to the lives of youth when many coping scales were designed in the 1960s (24). In addition, today’s youth are facing multiple social realities than past generations, mainly due to shifts in parents’ traditional roles. In particular, the increase in working mothers; the decline of the once-traditional nuclear family, with more unmarried couples sharing a home; the economic downturn; and a more sophisticated and demanding world impose novel forms of stress on youth. While it is possible that some youth will respond to these challenges by selecting traditional stress-coping mechanisms [social support, religion, etc. (25–27)], many may choose mechanisms involving electronic media (28). Therefore, an assessment scale that includes how youth use electronic media as a way to cope with stress is needed.

Even though electronic media is not mentioned specifically as a way of coping with stress in the classic empirical findings of Lazarus and Folkman (24), a scale that could embrace media exists: escape-avoidance. Briefly, the transactional model of stress and coping (24) allows the creation of a framework for evaluating and coping with stressful events based on person-environment transactions. When confronting a stressor, a person evaluates the potential threat and determines if the event is stressful, controllable, challenging, or irrelevant. Afterward, the person considers the available coping resources and options to confront the event. These include, among other strategies, moving away from the problem (escape-avoidance), pursuing social support, or accepting responsibility. The escape-avoidance scale included in most coping models includes questions related to coping with stress. Use of electronic media as a coping strategy could certainly fit in this scale, especially when we consider how much this pattern seems to be youth related. In this context, it is apparent that the actions of youth, who are often blamed for using electronic media to avoid in-person interactions, may have deeper meaning. They may be using media for entertainment, to avoid socializing with adults, or to cope with stress. Thus, we believe that including electronic media questions in coping assessments, such as those included in the escape-avoidance scale, will provide insight into the role that electronic media plays in the lives of youth.

Without a doubt, the digital era has created challenges and opportunities never before seen. Youth are now able to communicate and entertain themselves with portable phones, laptops, tablets, and/or consoles anywhere and anytime. There are undoubtedly many advantages associated with the amazing things people can do with technology. However, it would be shortsighted to consider these changes are all good or bad. Instead, we must remember Kranzberg’s first law of the role of technology: “technology is neither good nor bad – nor is it neutral” (29). To date, the use of electronic media by youth has not adequately been taken into account as a way to cope with stress, even though it is highly relevant. Here, we suggest a need for the development of a novel assessment scale that will facilitate studies of the role of electronic media as a youth coping strategy. Stress affects people differently, ranging from little or no effect to causing illness, including depression, and being a major trigger of chronic diseases (30–32). The way a person copes with stress is an important mediator of the stress response and outcome. Lifelong methods of coping with stress are established during youth, so it is especially important to learn more about the role of new factors that are particularly important to youths. Coping strategies related to electronic media should be considered and studied; as such strategies can affect how teens confront stressful events and might be related to the much-criticized use and abuse of technology gadgets by youth. With this in mind, we feel that there is a need for researchers to plan and develop coping scales that include electronic media as a means of coping for young people.

## Conflict of Interest Statement

The authors declare that the research was conducted in the absence of any commercial or financial relationships that could be construed as a potential conflict of interest.
